# Relative Performance and Predictive Values of Plasma and Dried Blood Spots with Filter Paper Sampling Techniques and Dilutions of the Lymphatic Filariasis Og4C3 Antigen ELISA for Samples from Myanmar

**DOI:** 10.3390/tropicalmed2020007

**Published:** 2017-04-11

**Authors:** Jesse Masson, Jan Douglass, Maureen Roineau, Khin Saw Aye, Kyi May Htwe, Jeffrey Warner, Patricia M. Graves

**Affiliations:** 1 College of Public Health, Medical and Veterinary Sciences, James Cook University, Cairns, QLD 4870, Australia; jan.douglass@my.jcu.edu.au (J.D.); Maureenroineau@wanadoo.fr (M.R.); jeffrey.warner@jcu.edu.au (J.W.); patricia.graves@jcu.edu.au (P.M.G.); 2 Department of Medical Research, Myanmar Ministry of Health and Sports, Nay Pyi Taw, Myanmar; ksadmr@gmail.com (K.S.A.); Kyimaywin31@gmail.com (K.M.H.)

**Keywords:** lymphatic filariasis, Og4C3, ELISA, filter paper, DBS, ICT, immunochromatographic test

## Abstract

Diagnostic testing of blood samples for parasite antigen Og4C3 is used to assess *Wuchereria bancrofti* in endemic populations. However, the Tropbio ELISA recommends that plasma and dried blood spots (DBS) prepared using filter paper be used at different dilutions, making it uncertain whether these two methods and dilutions give similar results, especially at low levels of residual infection or resurgence during the post-program phase. We compared results obtained using samples of plasma and DBS taken simultaneously from 104 young adults in Myanmar in 2014, of whom 50 (48.1%) were positive for filariasis antigen by rapid antigen test. Results from DBS tests at recommended dilution were significantly lower than results from plasma tested at recommended dilution, with comparisons between plasma and DBS at unmatched dilutions yielding low sensitivity and negative predictive values of 60.0% and 70.6% respectively. While collection of capillary blood on DBS is cheaper and easier to perform than collecting plasma or serum, and does not need to be stored frozen, dilutions between different versions of the test must be reconciled or an adjustment factor applied.

## 1. Introduction

Accurate and reliable screening assays that can be performed in low-resource laboratory settings have become vital for the Global Program to Eliminate Lymphatic Filariasis (GPELF). Currently, the use of the parasite antigen Og4C3 ELISA is among several techniques used to identify infection with lymphatic filariasis (LF) in *Wuchereria bancrofti* endemic areas [1,2,3]. Along with rapid immunochromatographic testing (ICT), which generates positive/negative results, the Og4C3 ELISA is used in monitoring and evaluation of the mass drug administration (MDA)-led elimination programs among endemic populations, producing quantitative results based on antigen units derived from a standard curve [4,5,6].

The Og4C3 ELISA, originally developed under Tropbio Pty. Ltd. at James Cook University, has been commercially available since the 1990s. Since 2013, the Og4C3 ELISA has been manufactured and supplied by Cellabs Pty. Ltd., Australia. Using categorical positive/negative results generated by using a cut-off value for positivity, the test has previously demonstrated a high level of sensitivity for the detection of filarial antigen when compared with the ICT rapid test [7,8,9]. Typically, the Og4C3 ELISA is performed using serum or plasma, either immediately after the sample has been obtained, or more often, from frozen samples. However, the Og4C3 ELISA also offers an alternative application method through the use of dried blood spots (DBS) collected on filter paper, an inexpensive and convenient method that requires less space and less stringent refrigeration for transport and storage.

Both plasma and DBS can make an important contribution to the final stages of LF elimination and to ensuring that re-emergence of disease has not occurred, despite some differences in practicability. For example, plasma samples can be directly applied to ELISA, yielding final results within a few hours if onsite laboratory facilities are available. However, DBS may be more convenient for field work and cost-effectiveness, as laboratory conditions are not mandatory despite the required overnight elution. Limited sample variation due to fluctuations in temperature and detection of antigen even after 12 months has also been demonstrated [10].

Despite the convenience in having two methods of application, studies which have directly compared Og4C3 ELISA DBS results to those from serum or plasma have shown discordance. Reeve and Melrose [11] compared results for 354 individuals from Papua New Guinea and observed significantly higher proportions of samples classified as positive by Og4C3 ELISA for serum (48.2%) than for DBS (32.5%). They demonstrated that the DBS technique yielded high specificity (99.2%; *N* = 391) and positive predictive value (PPV) (98.8%), but that sensitivity (67.2%; *N* = 354) and negative predictive values (NPV) (77%) were low when comparing DBS to serum as the gold standard. Interestingly, Tropbio and Cellabs recommend the dilution for plasma at 1:4 [12], while filter paper is recommended at an estimated dilution of 1:13.3, suggesting that the difference in dilution could be an underlying factor for discordance.

A second study by Itoh et al. [10], compared Og4C3 results for serum and DBS using a sample of 60 people in Sri Lanka, 55% of whom were reported to be positive for microfilariae, 65% positive by Og4C3 using serum, and 63.3% positive when using DBS. While these proportions positive by DBS and serum were not significantly different, this study used serum at a 1:3 dilution, applied at 16.7 μL per well, while DBS samples at an estimated dilution of 1:5 were applied at 3.3 μL per well. The authors then employed a 5-fold upward correction to the DBS antigen unit results to correct this difference in dilution. It is not clear how the results would have aligned if both sample types had been tested at currently recommended dilutions, and not adjusted.

Given the routine use of the Og4C3 ELISA by either plasma or DBS worldwide, this study aims to validate the existing instructions regarding the different dilutions of sample collected, and to determine if DBS is appropriate and comparable to plasma at the recommended dilutions.

## 2. Materials and Methods

The study site was the Amarapura Township of the Mandalay Region in Central Myanmar, an area known to have a high prevalence of LF infection. Persons aged 10–21 years were invited to participate. Ethical approval for the study was obtained from the James Cook University Research Human Ethics Committee, approval number H5261, and the Myanmar Ministry of Health and Sports. 

### 2.1. Study Population 

All participants were screened for LF infection using BinaxNOW^®^ filariasis ICT (Alere International Limited, Ireland). Participants who tested positive by ICT were age and gender matched to uninfected controls and invited to participate in the longitudinal study. Written consent was obtained from 18–21 year olds and from the parent or guardian of 10–17 year olds. Blood collection was conducted with the assistance of the Myanmar Ministry of Health and Sports.

### 2.2. Preparation of Plasma and DBS Samples

A 10 mL sample of venous blood was collected from each individual in cooled ethylenediaminetetraacetic acid (EDTA) anticoagulant vacutainers (BD Biosciences, Becton, Dickinson and Company, North Ryde, NSW, Australia). Ten microlitres of blood was blotted using a micropipette onto each of six protruding filter paper sections (TropBio filter papers), which were left to completely dry before being placed in individual plastic bags. The remaining whole blood was placed on crushed ice and delivered to the Public Health Laboratory in Mandalay within four hours of collection. Whole blood was spun for 15 min at 3000×*g* so that plasma could be aliquoted into 2 mL tubes. Plasma samples and DBS were stored short term at −20 °C until transported to Yangon on dry ice for −80 °C storage at the Department of Medical Research. One vial of each plasma sample was further aliquoted and refrozen before being shipped to James Cook University in Cairns, Australia, for storage at −80 °C. Filter papers were sealed in plastic containers and kept in either 4 °C refrigeration or hand luggage during direct transport to Cairns where they were stored at −80 °C.

### 2.3. Application of the TropBio Og4C3 Filariasis ELISA 

The Og4C3 antigen was detected in plasma using the commercially available TropBio Og4C3 kits (Cellabs Pty. Ltd., Manly, Australia) at 1:4 dilution (50 µL of plasma into 150 µL of sample diluent, rather than 100 µL of plasma into 300 µL of sample diluent as recommended by the TropBio kit, to conserve samples) and at 1:16 dilution (50 µL of the 1:4 dilution into 150 µL of sample diluent). DBS samples were not tested at 1:4, since the volume of the three DBS protrusions was too large to elute in a small enough volume to test at 1:4.

The DBS dilution used three filter paper protrusions per sample, representing 30 µL of whole blood. We diluted the DBS into 200 µL of diluent. If the haematocrit was 50%, this would give a dilution of 1:13, making the assumption that the dried blood occupies no volume. Given wide individual variation in haematocrit, we are not able to specify an exact dilution factor for DBS. The filter paper insert supplied by TropBio suggests that the DBS assay is approximately 4-fold less sensitive than plasma or serum tested at recommended dilution of 1:4. Therefore the dilution is likely in the range of 1:11 to 1:16. In this study all DBS were tested as per kit recommended dilution (three spots into 200 µL). DBS were eluted overnight at 4 °C.

Positive control samples of serum were created at identical dilutions to ensure plate-to-plate consistency using a pool of ten known positive sera from a LF endemic area of Papua New Guinea (PNG). Negative controls from Australian lab workers were also used. Diluted samples were boiled using a water bath for 10 min before centrifuging at 2000×*g* for 15 min. Fifty µL of the supernatant from each diluted sample was added to each well of the plate for primary sample incubation at 37 °C for 1 h, with the remainder of the ELISA technique performed as described in the instructions provided with the Tropbio Og4C3 kit [12]. Following initial incubation, wells which contained DBS samples were emptied and treated with washing buffer before the addition of 50 µL of 1% hydrogen peroxide solution, made by diluting 400 µL of 30% hydrogen peroxide in 11.6 mL of prepared wash buffer, and incubated for 10 min before subsequent washing.

### 2.4. Statistical Analysis

A standard curve was constructed for each ELISA plate using the kit-supplied controls, assigning an arbitrary high value of 32,768 units with subsequent 4-fold dilutions. The Softmax Pro v5 software (Molecular Devices, Sunnyvale, CA, USA) was used to convert optical density readings of the test samples to units based on a four-parameter curve. A single cut off point of >32 units (representing the sixth point of the standard curve from subsequent dilutions) was used to determine positive readings. Differences in unit values, between sampling techniques and test variations, were examined using paired *t*-tests, odds ratio, chi-square and correlation analysis. All analyses were performed using the IBM statistical software SPSS Version 23.0 (IBM, Armonk, NY, USA). Sensitivity, specificity, positive predictive value (PPV), and negative predictive value (NPV) of comparisons were also analysed. Confidence intervals (CI) were reported at 95%.

## 3. Results

Individuals between 10 to 21 years of age, irrespective of gender, were tested by ICT using fingerprick blood collected during October 2014. For each ICT-positive participant, an age and sex matched ICT negative participant was identified. Both were invited to return for full participation in a study of physical characteristics and to give a sample of venous blood for serological testing. Not all selected individuals (cases and controls) returned for the second venous sample; therefore, a final collection of 48 positive and 56 negative samples were included in this study.

### 3.1. Analysis of Categorical Results Used to Compare the ICT and Og4C3 ELISA 

To investigate how results classified as positive and negative varied between the ICT and the Og4C3 ELISA using plasma and DBS, categorical results were compared. Positive proportions of 46.2% obtained through the ICT were not significantly different to the proportion of 48.1% when using plasma at 1:4 dilution (Table 1). However, values became significantly different, dropping to 37.5% and 34.6% respectively, when plasma at 1:16 dilution or DBS was used (Table 1). 

Holding the ICT as a relative standard, agreement with the Og4C3 ELISA using plasma and DBS was assessed. When ELISA using plasma at a 1:4 dilution was compared to ICT, sensitivity was 85.4% and specificity 83.9%, while PPV and NPV were 82.0% and 87.0%, respectively (Table 2). The use of a 1:16 dilution for plasma or use of DBS resulted in a decrease in sensitivity and NPV when compared to ICT, while specificity and PPV increased (Table 2). This suggests that the Og4C3 ELISA is significantly affected by dilution and application method when compared to ICT, with the 1:4 dilution yielding higher sensitivity and NPV, while the 1:16 plasma dilution or DBS yields higher specificity and PPV.

Odds ratios were used to predict the likelihood of positive and negative results from comparative Og4C3 samples when compared to the ICT standard. Odds ratio was highest at 90.8 for comparison against plasma using a 1:16 dilution, but decreased to 30.6 when using plasma at 1:4 dilution, and to 38.9 when using DBS (Table 2). This suggests that agreement between the ICT and Og4C3 ELISA is best when using the Og4C3 with plasma at a 1:16 dilution.

### 3.2. Analysis of Categorical Results Used to Compare Plasma at 1:4 and 1:16 Dilutions and DBS

To assess results achieved by Og4C3 ELISA, plasma application at a 1:4 dilution was held as a standard for comparison. Again, we used chi-square tests to find a significant value of 7.5 (*p* = 0.009) when comparing plasma at 1:4 to DBS, which remained significant at 5.3 (*p* = 0.04) when plasma was compared using 1:4 and 1:16 dilutions (Table 3). Therefore, both plasma application at 1:16 and DBS give different results to plasma tested at the recommended 1:4 dilution.

To investigate if matched dilutions might improve plasma and DBS concordance between proportions, both methods were compared using plasma at 1:16 as the new standard. The difference was not significant when comparing plasma at 1:16 and DBS, achieving a chi-square value of 0.7 (Table 4). This confirms high agreement of positive proportions when plasma and DBS are applied using similar dilutions.

To determine how application of plasma and DBS with recommended dilution affects final results of each test, sensitivity, specificity, PPV and NPV were calculated. Sensitivity and NPV were low at 60.0% and 70.6% when DBS was compared to plasma at 1:4 (Table 5). Higher values of 88.9% and 83.3% were recorded for specificity and PPV respectively (Table 5). Test performance remained relatively unchanged when plasma at 1:16 was compared to plasma at 1:4, with the exception of an increase in sensitivity to 66.0% over DBS when using a similar dilution (Table 5).

Kappa agreement statistic was used to analyse concordance between comparisons against the 1:4 diluted plasma gold standard. Kappa values were relatively low at 0.55 and 0.49 for comparisons of plasma at 1:4 with plasma at 1:16 dilution and DBS respectively.

To assess if more closely-matched dilutions might improve positive and negative detection rates, plasma at 1:16 dilution was employed as a standard and compared against DBS. This resulted in relatively high sensitivity (79.5%), specificity (92.3%), PPV (86.1%), and NPV (88.2%), suggesting that both plasma and DBS yield highest concordance in positive and negative results when applied using similar dilutions (Table 6).

An odds ratio of 46.5 was also achieved when comparing plasma at a 1:16 dilution and DBS (Table 6) which was higher than odds ratio achieved when using plasma at 1:4 dilution as the standard (Table 5). This suggests a stronger association between plasma and DBS when dilutions are matched.

Concordance between the 1:16 diluted plasma sample and the DBS sample was also measured using kappa agreement statistic. Values were higher at 0.73 than observed with comparisons that used a 1:4 diluted gold standard. This suggests higher concordance between samples when dilutions are matched.

### 3.3. Analysis of Quantitative Mean Results Used to Compare Plasma at 1:4 and 1:16 Dilutions and DBS

**Figure 1:**
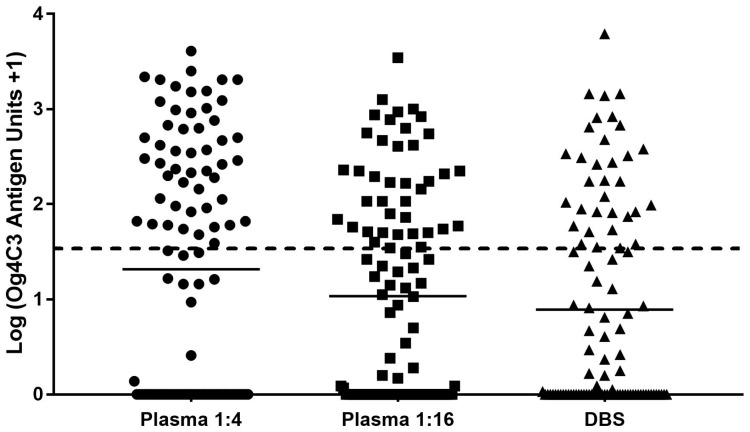
Filariasis Og4C3 ELISA antigen unit concentration comparisons of dilution, plasma and whole blood application. Horizontal black lines refer to mean log antigen unit concentration of each assay (Table 7). All antigen unit concentrations above the defined cut-off (log (32 antigen units + 1)), shown by the horizontal segregated line, are considered positive, while all readings below are considered negative.

Antigen unit values are shown for each sample type and dilution expressed as log (units+1) in Figure 1.

Analysis of log unit values + 1 was used to approximate a normal distribution for comparisons. From these units, the mean log value for plasma at 1:4 (1.32) was significantly higher than the mean log values achieved for DBS (0.89; *p* < 0.0001) (Table 7). The mean log for plasma at 1:4 was also significantly higher than for plasma at 1:16 dilution (Table 7). Despite a statistically significant *p* value when dilutions were similar for plasma and DBS, the mean difference was smaller than previous comparisons. These quantitative comparisons are consistent with the results described above. 

### 3.4. Correlation Comparisons Analysis of Og4C3 Concentrations from Plasma and Eluates from DBS 

**Figure 2:**
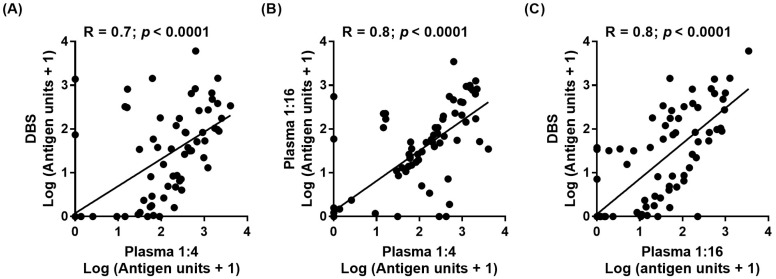
Filariasis Og4C3 ELISA antigen unit concentration correlation comparisons. (**A**) Plasma at recommended 1:4 dilution plotted against DBS samples. (**B**) Plasma at recommended 1:4 dilution plotted against 1:16 dilution. (**C**) Plasma at 1:16 dilution plotted against DBS samples.

To compare differences in antigen unit concentrations between tests, correlation coefficients were measured between sample types and dilutions. When comparing recommended dilutions of plasma at 1:4 against DBS, correlation was lowest at 0.7 (Figure 2A). Correlation increased to 0.8 when plasma at 1:4 and 1:16 dilutions were compared (Figure 2B), and when plasma at 1:16 and DBS were compared (Figure 2C). This suggests improved correlation is achieved through either matched sample application or dilution.

## 4. Discussion

The major finding of this study is that the agreement of results between plasma and DBS samples is lowest when using recommended dilutions of 1:4 for plasma, and that better agreement between these tests is achieved when using plasma at 1:16 against DBS. However, significantly fewer samples are classed as positive when using the lower 1:16 dilution for plasma or using DBS.

We showed that the ICT had similar positive proportions and predictive values to the Og4C3 ELISA when using plasma at 1:4, but that sensitivity is reduced when using the 1:16 dilution. Previous literature has shown that the ICT is less sensitive than the Og4C3 ELISA at 1:4 dilution, detecting only 66.5% of known bancroftian filariasis cases with no microfilariae confirmed by microscopy [13], but that both tests yield identical detection rates when microfilariae were detectable [13,14,15,16]. Og4C3 has also been reported to detect significantly smaller titres of filarial antigen when compared to the ICT [13,17], although ICT and Og4C3 both showed an improved detection rate average of up to 25% over microfilariae detection[18]. The current study suggests that despite the higher sensitivity of Og4C3 at lower antigen dilutions, the dilution recommended for DBS application reduces the amount of antigens per sample to a titre that will not accurately yield a positive result when the disease is present.

When analysing positive proportions, results were similar at 46.15% and 48.08% for ICT and plasma at 1:4, and again at 37.50% and 34.62% for plasma at 1:16 and DBS. Positive proportion similarity between plasma and DBS has previously been discussed by Itoh and colleagues [10] as having a 97.4% concordance. However, these results were produced using a 5-fold upwards adjustment to the DBS antigen units.

Lack of concordance between plasma at 1:4 and DBS was supported by Chi-square analysis, which showed significant difference when recommended dilutions for plasma and DBS were used, but no significant difference with matched dilution comparisons. These data support the notion that it is not sample type per se but dilution factor that affects quantitative results, and lower positive proportions for plasma at 1:16 and DBS are detected when compared to ICT or plasma at 1:4 dilution.

Sensitivity and NPV was lowest when DBS was compared to plasma using recommended dilutions. This comparison also yielded an odds ratio of 12.00 and kappa of 0.49, the lowest value among each of these comparisons. These results are similar to those found by Reeve and Melrose [11], where sensitivity and NPV was 67.2% and 77.0% respectively using the dilutions recommended by the Og4C3 kit. As sensitivity and NPV increased to 79.49% and 88.24% respectively when comparing plasma at 1:16 and DBS samples, these results suggest that dilution directly affects how likely it is that the assay will detect the presence of *Wuchereria bancrofti* in someone with LF, as well as how likely it is to detect someone with a negative result when they do not have LF. These statistics were also supported by an odds ratio, which was highest at 45.50, and by the kappa agreement statistic, which was also highest at 0.73, when dilutions were matched.

There was a higher mean antigen concentration in plasma compared to DBS at recommended dilutions, but the difference in mean antigen units was greatly reduced when dilutions were more closely matched. While plasma samples were tested at 1:4 and 1:16 dilutions, equal to a 4-fold difference, geometric mean (antigen unit +1) values were 19.75 and 9.83 respectively. The difference in means is much less than 4-fold. The geometric means were 9.83 and 6.81 for plasma at 1:16 and DBS respectively. The non-linear antigen unit relationship to dilution factor may be attributed to high saturation of each ELISA well by a higher concentration of sample, which would be left with fewer spare antibodies bound to the plastic surface available for the free floating antigens to bind during the initial 1-h incubation period. It is possible that a lower concentration of sample antigen may bind fewer antibodies initially, but will not saturate the well as the higher concentration sample would, allowing a greater number of unbound antibodies on the plate to attach to free floating antigens during incubation [19,20].

Correlation was found to be highest when either dilutions or sample application methods were matched, but was low when plasma at 1:4 and DBS were compared. No previous study has compared matching low dilutions but it is not surprising that matched dilutions may result in higher agreeability between plasma and DBS. This is important as higher dilutions are less sensitive overall when compared with ICT and therefore must be used with caution.

The results of this study indicate that the currently recommended dilution for the Og4C3 ELISA using DBS is not as sensitive as for plasma, and that more than 25% of positive cases may be being missed when using DBS. Concordance between methods would improve if similar dilutions were used. Unfortunately, a 1:4 dilution is not possible with the current prescribed method of DBS preparation and amount of sample, since that would require eluting three filter paper discs in 60 μL of diluent, leaving insufficient supernatant for a single well after boiling and spinning. While high correlation can be achieved when the fold difference in antigens is matched [10], Lalitha and colleagues [21] show that correlation can drop to 0.83 when plasma and DBS are used at 1:4 and 1:20 dilutions respectively.

The outcomes of this research lead to a recommendation that further assessment be carried out to determine how both plasma and DBS perform at other matched dilutions, such as 1:8 or 1:10. Any such tests must accommodate the volume needed to fully saturate each dried blood spot, allow for boiling, precipitation and spinning, and yielding 50 μL to be applied to the well of an Og4C3 ELISA. Improved concordance between Og4C3 plasma and DBS application that also achieves a similar detection rate as the ICT would greatly support the reliability of the Og4C3 ELISA for field collection in non-laboratory locations, as DBS is a convenient method of collecting samples, requiring less in-depth training, and less storage space or refrigeration during transport.

A major limitation attributed to enzyme immunoassays, referred to as the ‘hook’ effect, is shown when a decrease in reactivity is seen as the concentration of free antigen or antibody increases [22,23,24]. As each plate will always give different readings, a reliable standard curve must be used to mitigate plate-to-plate variation and avoid error in classifications of positive or negative from test to test. One strategy may be to perform each sample at two dilutions in order to obviate such occurrences and to ensure that antibody or antigen excess does not occur. A two-dilution application could also evaluate each individual plate by showing if any non-linearity between results has occurred.

In conclusion, the current recommended dilutions for plasma and DBS did not demonstrate adequate agreement. The data obtained shows that both plasma and DBS are capable of yielding similar results, but only when dilutions are more closely matched than is currently recommended. Definition of a single suitable dilution factor lower than 1:16 which can be applied to both DBS and plasma assays, and to compare specificity, sensitivity and predictive values between DBS and plasma upon application is required. Results achieved from antigen unit analysis using DBS assays need to be adjusted upwards to be comparable with plasma tests. We recommend that similar studies be repeated in other endemic situations, with different test predictive values, to confirm our results and to determine the adjustment factors required. In the longer term, the dilutions for the two test types need to be reconciled.

## Figures and Tables

**Table 1:** Positive proportions and chi-square results of comparisons between the immunochromatographic test and the Og4C3 ELISA.

Standard	N Positive	% Positive (95% Confidence Interval (CI))	Comparative Test	N Positive	% Positive (95% CI)	McNemar Chi Sq	*P* Value
ICT	48	46.2 (36.9–55.7)	Plasma 1:4	50	48.1 (38.7–57.6)	0.3	*p* = 0.8
-	-	-	Plasma 1:16	39	37.5 (28.8–47.1)	6.2	*p* = 0.02
-	-	-	Dried blood spot (DBS)	36	34.6 (26.2–44.2)	8.00	*p* = 0.008

**Table 2:** Sensitivity, specificity, positive predictive value (PPV), negative predictive value (NPV) and odds ratio results from ICT and Og4C3 ELISA comparisons.

Standard	Comparative Test	Sensitivity (95% CI)	Specificity (95% CI)	PPV (95% CI)	NPV (95% CI)	Odds Ratio (95% CI)	*P* Value
ICT	Plasma 1:4	85.4% (72.2–93.9)	83.9% (71.7–92.4)	82.0% (71.2–89.3)	87.0% (77.0–93.1)	30.6 (10.5–89.4)	*p* <0.0001
-	Plasma 1:16	77.1% (62.7–88.0)	96.4% (87.7–99.6)	94.9% (82.5–98.6)	83.1 (74.5–89.2)	90.8 (19.0–433.8)	*p* <0.0001
-	DBS	68.8% (53.8–81.3)	94.5% (85.1–98.9)	91.7% (78.3–97.1)	77.9% (69.8–84.4)	38.9 (10.5–144.6)	*p* <0.0001

**Table 3:** Positive proportions and chi-square results of comparisons between Og4C3 ELISA application method and dilution.

Standard	N Positive	% Positive (95% CI)	Comparative Test	N Positive	% Positive (95% CI)	McNemar Chi Sq	*P* Value
Plasma 1:4	50	48.1 (38.7–57.6)	DBS	36	34.6 (26.2–44.2)	7.5	*p* = 0.009
-	-	-	Plasma 1:16	39	37.5 (28.8–47.1)	5.3	*p* = 0.04

**Table 4:** Positive proportions and chi-square results of comparisons between Og4C3 ELISA applications at 1:16 dilution.

Standard	N Positive	% Positive (95% CI)	Comparative Test	N Positive	% Positive (95% CI)	McNemar Chi Sq	*P* Value
Plasma 1:16	39	37.5 (28.8–47.1)	DBS	36	34.6 (26.2–44.2)	0.7	*p* = 0.6

**Table 5:** Sensitivity, specificity, PPV, NPV and odds ratio results from Og4C3 method and dilution comparisons.

Standard	Comparative Test	Sensitivity (95% CI)	Specificity (95% CI)	PPV (95% CI)	NPV (95% CI)	Odds Ratio (95% CI)	*P* Value
Plasma 1:4	DBS	60.0% (45.2–73.6)	88.9% (77.4–95.8)	83.3% (67.2–93.6)	70.6% (58.3–81.0)	12.0 (4.3–33.3)	*p* <0.0001
-	Plasma 1:16	66.0% (51.2–78.8)	88.9% (77.4–95.8)	84.6% (69.5–94.1)	73.9% (61.5–84.0)	15.5 (5.5–43.5)	*p* <0.0001

**Table 6:** Sensitivity, specificity, PPV, NPV and odds ratio results from Og4C3 ELISA applications of plasma at 1:16 dilution and DBS.

Standard	Comparative Test	Sensitivity (95% CI)	Specificity (95% CI)	PPV (95% CI)	NPV (95% CI)	Odds Ratio (95% CI)	*P* Value
Plasma 1:16	DBS	79.5% (63.5–90.7)	92.3% (83.0–97.5)	86.1% (70.5–95.3)	88.2% (78.1–94.8)	46.5 (14.0–154.2)	*p* < 0.0001

**Table 7:** Comparison of mean Og4C3 antigen units between sample applications and dilutions.

Group 1			Group 2			*t*-Test			
Test	Mean Log (Antigen Unit +1)	Geometric Mean	Test	Mean Log (Antigen Unit +1)	Geometric Mean	N	Mean Log Difference	SD	*P* Value
Plasma 1:4	1.32	19.75	DBS	0.89	6.81	104	0.42	0.88	*p* <0.0001
Plasma 1:4	1.32	19.75	Plasma 1:16	1.03	9.83	104	0.28	0.74	*p* <0.001
Plasma 1:16	1.03	9.83	DBS	0.89	6.81	104	0.14	0.67	*p* = 0.03
